# Etiologic diagnosis of hospitalized patients with community-acquired pneumonia: bridging the evidence gap

**DOI:** 10.36416/1806-3756/e20260206

**Published:** 2026-05-22

**Authors:** Filipe Amado, Jorge Ibrain Figueira Salluh, Thales Teixeira, Maria Eduarda de Oliveira Novaes, Glória Martins, Caroline Correa, Melissa Tassano Pitrowski, Bruno Adler Maccagnan Pinheiro Besen, Antonio Paulo Nassar, Pedro Manuel Sarmento Rodrigues Povoa, Cassia Righy Shinotsuka, Giulliana M. Moralez

**Affiliations:** 1. Instituto D’Or de Ensino e Pesquisa - IDOR - Rio de Janeiro (RJ) Brasil.; 2. Programa de Pós-Graduação em Medicina Interna, Universidade Federal do Rio de Janeiro - UFRJ - Rio de Janeiro (RJ) Brasil.; 3. Comprehensive Health Research Centre - CHRC - NOVA Medical School, Universidade NOVA de Lisboa, Lisboa, Portugal.; 4. Departamento de Cuidados Intensivos, Hospital Copa Star. Rio de Janeiro (RJ) Brasil.; 5. Unidade de Terapia Intensiva, AC Camargo Cancer Center. São Paulo (SP) Brasil.; 6. Unidade de Cuidados Intensivos Polivalentes, Hospital de São Francisco Xavier - CHLO - Lisboa, Portugal.; 7. Instituto Nacional de Infectologia Evandro Chagas, Fundação Oswaldo Cruz, Rio de Janeiro (RJ) Brasil.; 8. Universidade do Estado do Rio de Janeiro - UERJ - Rio de Janeiro (RJ) Brasil.

## INTRODUCTION

Community-acquired pneumonia (CAP) remains one of the leading causes of hospitalization and mortality worldwide, particularly in low- and middle-income countries (LMICs) such as Brazil, resulting in a substantial economic burden on healthcare systems. While *Streptococcus pneumoniae* remains the most commonly identified bacterial pathogen, contemporary epidemiological studies have been documenting a growing contribution of respiratory viruses, most strikingly underlined by the COVID-19 pandemic, as well as gram-negative enteric bacilli, polymicrobial infections, and mixed viral-bacterial coinfections.^1^ ^,^ ^2^

Nevertheless, determining CAP etiology is challenging given the complex interplay of geographic and population-related factors, including ageing populations, immunosuppression, and evolving vaccination coverage, as well as seasonality, prior antimicrobial use, and the limited availability of diagnostic methods. In Brazil, as in most LMICs, this challenge is compounded by a disproportionately small evidence base, as the bulk of the data informing international guidelines derives from high-income countries, a fundamental mismatch that this viewpoint aims to address.^3^ ^,^ ^4^

Recent guidelines highlight the importance of early and timely use of appropriate antimicrobials, especially in patients with sepsis. In this setting, the choice of initial treatment is often empirical (based on the prevalence of the most frequent pathogens). In this context, identifying the etiology of CAP is fundamental to guiding antibiotic therapy and may improve outcomes at three levels: clinical, through lower adverse event rates and mortality; ecological, through reduced selection of multidrug-resistant organisms; and economic, through lower antibiotic costs and potentially shorter length of hospital stay. Identifying the causative pathogen, therefore, represents both a clinical and a stewardship imperative that remains systematically underachieved in Brazil and across LMICs.^5^

## CURRENT GUIDELINE RECOMMENDATIONS FOR ETIOLOGIC DIAGNOSIS: INITIAL MANAGEMENT AND DIAGNOSTIC WORK-UP

Current guidelines recommend a stepwise diagnostic strategy built on three pillars: prompt collection of high-quality microbiological samples before antibiotic initiation; integration of conventional cultures with selective use of rapid molecular tests; and systematic reassessment when treatment failure occurs or alternative diagnoses emerge.

The 2019 American Thoracic Society and Infectious Diseases Society of America (ATS/IDSA) and the 2023 international European Respiratory Society, European Society of Intensive Care Medicine, European Society of Clinical Microbiology and Infectious Diseases, and Latin American Thoracic Association guidelines (ERS/ESICM/ESCMID/ALAT) recommend a structured approach to perform the etiologic diagnosis of hospitalized patients with CAP, particularly in severe cases.^6^ ^,^ ^7^

A key principle is the prompt collection of good-quality microbiological samples before initiating empiric antibiotic therapy whenever possible, namely, blood cultures and lower respiratory tract samples. Although there is limited evidence of the clinical benefit from blood cultures as well as a low positivity rate (10-20%), the identification of a pathogen improves the appropriateness of antibiotic therapy.^8^ Concerning the type of respiratory samples, the quality increases from sputum to a distal sample collected with bronchoscopy. Specimens such as endotracheal aspirates and BAL are preferred over upper respiratory tract samples, particularly in critically ill or mechanically ventilated patients.^9^ These lower respiratory tract samples better reflect the site of infection and increase the likelihood of identifying causative pathogens.

In hospitalized patients with CAP, the guidelines strongly recommend obtaining gram staining and conventional culture of respiratory secretions.^7^ In addition, the cytologic examination of the respiratory sample is useful, since a neutrophil count > 50-65% is suggestive of the presence of pneumonia.^10^

The 2023 ERS/ESICM/ESCMID/ALAT guidelines recommend, whenever available, rapid molecular diagnostic techniques, especially multiplex PCR, as an adjunct to conventional microbiological testing. In addition, pathogen-specific biomarkers are very useful since they could identify specific pathogens in a couple of hours with an impact on empiric antibiotic therapy.^11^ Rapid antigen tests, with variable diagnostic performance, for influenza and COVID19 are very easy to perform and have a very short response time (< 15 min). Besides that, there are also pneumococcal and *Legionella* sp. urinary antigen tests. Although *Legionella* sp. is a rare pathogen in CAP, it is usually associated with outbreaks. A positive urinary pneumococcal antigen test strongly suggests a pneumococcal infection.

Despite advances in diagnostic tools, important limitations remain. CAP continues to pose diagnostic challenges due to the presence of non-infectious conditions that can mimic pneumonia, such as inflammatory or autoimmune lung diseases. In such cases, failure to respond to treatment should prompt reconsideration of the diagnosis.^10^

## DIAGNOSTIC METHODS

The main diagnostic recommendations aimed at identifying etiological agents involve ordering pathogen-specific biomarkers, such as urinary antigen tests for pneumococcus and legionella, in addition to blood cultures, sputum culture, tracheal aspirate culture, or samples obtained by bronchoscopy (BAL, if feasible), PCR for respiratory viruses, rapid influenza and SARS-CoV-2 antigen tests, and nasal methicillin-resistant *Staphylococcus aureus* screening when indicated.^12^

The sensitivity of the urinary antigen test for pneumococcus ranges from 50% to 80% and its specificity exceeds 90%.^7^ This test has significant clinical importance, because *S. pneumoniae* is the most commonly identified pathogen in cases of CAP with a defined bacterial etiology. A positive pneumococcal urinary antigen test strongly suggests pneumococcal infection, pneumonia being the most common clinical manifestation.^11^ In turn, the urinary antigen test for legionella shows sensitivity between 70% and 100% and specificity between 95% and 100%.^13^ A positive result justifies modification of antibiotic therapy, whereas a negative result suggests the absence of recent or active legionella infection or the presence of a strain different from serogroup 1.

The development of new methods for microbiological identification has enabled earlier results.^13^ Examples such as multiplex PCR^14^ and matrix-assisted laser desorption/ionization time-of-flight mass spectrometry represent significant advances. The latter allows rapid and accurate identification of microorganisms (fungi or bacteria), reducing by up to 24 h the time required to obtain microbiological diagnosis in comparison with conventional systems.^15^ Multiplex PCR panels are molecular tests that enable the simultaneous detection of multiple respiratory pathogens and resistance genes from a single sample. These panels identify a wide range of viruses (such as influenza, respiratory syncytial virus, rhinovirus, and coronavirus) and bacteria (such as *S. pneumoniae*, *Mycoplasma pneumoniae*, and *Legionella pneumophila*), showing high sensitivity and specificity.^16^

Despite its low cost, the use of the pneumococcal urinary antigen test remains limited in clinical practice. In a higher-cost scenario, and therefore not always widely accessible, the use of molecular methods carries the risk of detecting colonizing or non-infectious organisms, which may also lead to overtreatment.^17^ Potential risks associated with multiplex PCR testing include costs and the possibility of inappropriate escalation of antibiotic therapy due to false-positive results.

**Figure 1:**
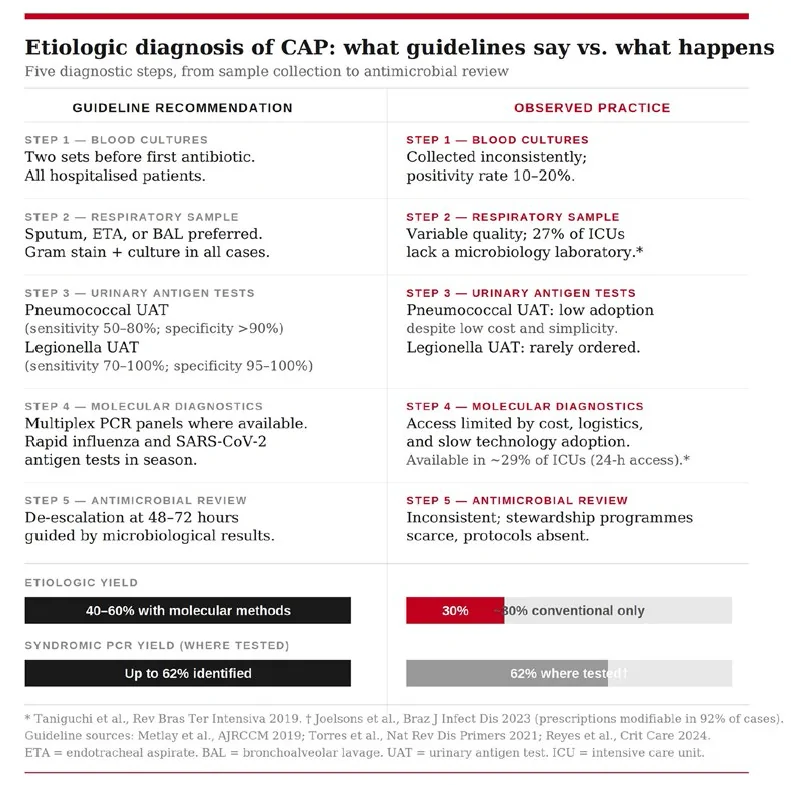
Etiologic diagnosis of community-acquired pneumonia (CAP): what guidelines say vs. what really happens. ETA: endotracheal aspirate; and UAT: urinary antigen testing.

Diagnostic practices in severe pneumonia differ significantly between high-income countries and regions such as Brazil and Latin America.^18^ In developed countries, there is greater access to methods such as molecular testing, although etiological identification rates remain limited, with frequent use of empirical antibiotic therapy based on clinical criteria. In contrast, in LMIC, infrastructure constraints, limited availability of diagnostic tests, and low adherence to stewardship programs further hinder microbiological identification and favor inappropriate antimicrobial use, with potential negative impact on outcomes (Figure 1).

## IMPLEMENTATION BARRIERS AND SOLUTIONS FOR LIMITED RESOURCE SETTINGS

Contemporary CAP guidelines recognize the etiological diagnosis as a core component of high-quality care. Identifying pneumonia-related pathogens informs decision-making at three levels: 1) at the patient level, it supports appropriate therapy, facilitating both escalation when needed and safe de-escalation when possible; 2) at the local health system level, it helps define institutional pathogen distribution and resistance patterns, allowing empirical treatment protocols to reflect the local epidemiology better; and 3) at the regional and national levels, etiological investigations strengthen surveillance, detect outbreaks, detect changing microbial trends, and support public health policies.

Why, then, has this approach not been systematically incorporated into hospital practices? One explanation is that diagnostic capacity remains unevenly distributed and is often insufficient for broad implementation. The global survey-based study (D-PRISM) highlighted marked disparities across income settings in the availability and use of diagnostic tools in the ICU. In LMICs, bronchoscopy was reported as available by 61% of respondents, but only 29% had 24-hour access.^18^ Costs, logistic challenges, and slow technology adoption are access barriers to test execution (such as multiplex PCR syndromic panels). In a Brazilian survey including 277 ICUs, 26.8% of those did not have a microbiology lab structure, which was associated with limited access to broad-spectrum antibiotics and worse outcomes.^19^

However, in places where resources are available, a limited implementation of guidelines may also be undermined by the lack of standardized protocols, insufficient training, delays in transport and processing, poor communication between laboratory and clinical teams, and uncertainty about how the results should modify antimicrobial prescriptions.

The use of syndromic PCR testing for critically ill patients with pneumonia is associated with more adequate initial antimicrobial therapy and shorter time to effective treatment, but not with lower in-hospital mortality.^20^ Brazilian data showed that a multiplex PCR panel identified an etiologic agent in 61.5% of hospitalized patients, far exceeding the yield of conventional methods alone, and the authors estimated that prescriptions could have been modified in 92.3% of cases, mostly through antibiotic de-escalation and more rational antiviral use.^21^ Thus, the challenge is not only to expand testing but also to ensure that the results would be actionable within stewardship-oriented clinical pathways.

Solutions for limited-resource settings should address these specific barriers. Where infrastructure is lacking, it is important to prioritize strengthening the laboratory base, ensuring minimum microbiology capacity, improving specimen logistics, and expanding trained human resources. Where infrastructure exists, the focus should be on protocolizing, monitoring turnaround time, clinician education, stewardship integration, and feedback loops linking lab results to antimicrobial review at 48-72 h.^22^

## RESEARCH PRIORITIES

Advancing the etiologic diagnosis of CAP for hospitalized patients in Brazil, as well as in other LMICs, requires a coordinated research agenda that spans evidence synthesis, infrastructure mapping, primary data generation, and implementation. We propose seven interrelated priorities.

**Figure 2:**
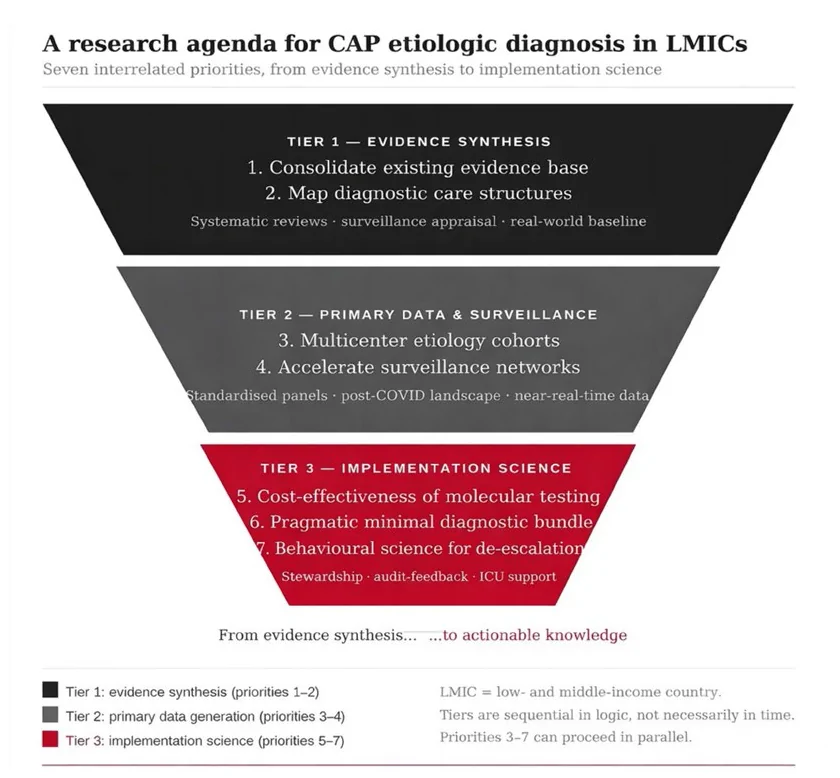
A research agenda for community-acquired pneumonia (CAP) etiologic diagnosis in low- and middle-income countries (LMICs).

1. Consolidating the existing evidence base. A clearer picture of what is already known and, more importantly, what is missing should be built through systematic reviews and meta-analyses focused on national and regional data from LMICs, complemented by a structured appraisal of national surveillance systems (i.e., SIVEP-Gripe, SINAN, and InfoGripe) and other existing clinical registries.^23^ ^,^ ^24^ This foundational step is essential to avoid redundant efforts and to identify geographic, seasonal, and demographic blind spots that should shape subsequent studies.
2. Mapping the structure and process of diagnostic care. Before generating new evidence, we need to understand the real-world diagnostic workup for CAP across hospitals (ICUs and emergency departments). A mapping exercise should describe both structural availability (microbiology laboratories, molecular platforms, turnaround times) and process-of-care measures (Are evidence-based protocols available? When, to whom, and how do results influence prescribing patterns?). Without this baseline, interventions risk being designed for an idealized rather than actual clinical environment.
3. Performing large multicenter etiology studies in Brazil and other LMICs. Contemporary, multicenter cohorts using standardized case definitions and a comprehensive diagnostic panel, including molecular platforms, are needed. Such studies should capture regional heterogeneity, seasonal dynamics, and the evolving post-pandemic microbiological landscape, improving the setting adaptation of current recommendations of guidelines. Such studies would provide the empirical foundation for national and regional guidelines and help to guide empirical therapy recommendations tailored to the current epidemiology.
4. Integrating and accelerating surveillance networks. Existing surveillance platforms should be interconnected to enable near-real-time availability of local microbiological and syndromic patterns. Faster data processing and open dashboards at the regional level would allow clinicians to adjust empirical therapy based on current circulating pathogens, transforming surveillance from a retrospective epidemiologic tool into an actionable bedside resource.
5. Evaluating cost-effectiveness of molecular diagnostics in resource-limited settings. Making etiologic diagnosis clinically useful requires modernized microbiology laboratories, auditable standardized procedures, and effective integration of results into electronic health record and decision support tools. The incorporation of multiplex PCR and syndromic panels in LMICs must be evaluated not only in terms of diagnostic yield but also cost-effectiveness, antimicrobial stewardship impact, and clinically relevant outcomes.
6. Evaluating a pragmatic “resource-limited” diagnostic bundle. In parallel with high-complexity approaches, there is a pressing need to define and test minimal diagnostic bundles, for example, blood cultures combined with urinary antigen testing and, in the due season, where relevant, rapid influenza assays. Studies should assess whether such pragmatic strategies offer a reasonable compromise among diagnostic performance, feasibility, and costs in settings where molecular diagnostics remain inaccessible (Figure 2).
7. Using behavioral analysis and implementation science for antimicrobial de-escalation. Even when etiologic diagnosis is achieved, de-escalation remains inconsistent. Research informed by behavioral analysis and implementation science should identify the cognitive, institutional, and workflow barriers that prevent clinicians from acting on diagnostic information, and should test interventions, such as audit and feedback, decision support, and stewardship-led rounds in ICUs adapted to the local settings.

Together, these priorities define a pragmatic and achievable research program designed to transform not only the understanding but the management of CAP etiologic diagnosis in Brazil and across LMICs. The evidence gap is real, its clinical consequences measurable, and the tools to address it are increasingly within reach.
